# Pediatric Atypical Hemolytic Uremic Syndrome Advances

**DOI:** 10.3390/cells10123580

**Published:** 2021-12-18

**Authors:** Rupesh Raina, Nina Vijayvargiya, Amrit Khooblall, Manasa Melachuri, Shweta Deshpande, Divya Sharma, Kashin Mathur, Manav Arora, Sidharth Kumar Sethi, Sonia Sandhu

**Affiliations:** 1 Akron Nephrology Associates/Cleveland Clinic Akron General Medical Center, Akron, OH 44307, USA; nina.vijayvargiya@emory.edu (N.V.); amritkhoob@gmail.com (A.K.); shweta.deshpande96@gmail.com (S.D.); kashinmathur@gmail.com (K.M.); manavarora105@gmail.com (M.A.); 2 Department of Nephrology, Akron Children’s Hospital, Akron, OH 44308, USA; 3 Department of Medicine, Northeast Ohio Medical University, Rootstown, OH 44272, USA; mmelachuri@neomed.edu (M.M.); dsharma@neomed.edu (D.S.); 4 Pediatric Nephrology & Pediatric Kidney Transplantation, Kidney and Urology Institute, Medanta, The Medicity Hospital, Gurgaon 122007, India; sidsdoc@gmail.com; 5 Hematology and Oncology, Cleveland Clinic Akron General Medical Center, Akron, OH 44307, USA; sandhuS4@ccf.org

**Keywords:** pediatric aHUS, aHUS advancements

## Abstract

Atypical hemolytic uremic syndrome (aHUS) is a rare disorder characterized by dysregulation of the alternate pathway. The diagnosis of aHUS is one of exclusion, which complicates its early detection and corresponding intervention to mitigate its high rate of mortality and associated morbidity. Heterozygous mutations in complement regulatory proteins linked to aHUS are not always phenotypically active, and may require a particular trigger for the disease to manifest. This list of triggers continues to expand as more data is aggregated, particularly centered around COVID-19 and pediatric vaccinations. Novel genetic mutations continue to be identified though advancements in technology as well as greater access to cohorts of interest, as in diacylglycerol kinase epsilon (DGKE). DGKE mutations associated with aHUS are the first non-complement regulatory proteins associated with the disease, drastically changing the established framework. Additional markers that are less understood, but continue to be acknowledged, include the unique autoantibodies to complement factor H and complement factor I which are pathogenic drivers in aHUS. Interventional therapeutics have undergone the most advancements, as pharmacokinetic and pharmacodynamic properties are modified as needed in addition to their as biosimilar counterparts. As data continues to be gathered in this field, future advancements will optimally decrease the mortality and morbidity of this disease in children.

## 1. Introduction

Atypical hemolytic uremic syndrome (aHUS), characterized by a triad of thrombocytopenia, acute kidney injury (AKI), and microangiopathic hemolytic anemia, is a rare form of thrombotic microangiopathy (TMA) caused by dysregulation of the alternative pathway (AP) [1]. Clinical diagnosis of aHUS can be challenging as it relies on the symptomatic recognition of TMAs, yet it requires excluding all other causes of TMAs and HUSs. Thus, the precise incidence of aHUS in pediatric and young adult populations (<20 years of age) is fluid in nature. Typically diagnosed at 2 years of age, aHUS is associated with a mortality rate of 20–25% and a morbidity of 48%, as pediatric patients typically progress to end stage renal disease (ESRD) [1,2].

Genetic variants in complement regulatory proteins (CRPs) account for 50% to 60% of all aHUS cases, with approximately 30–50% having no known identifiable mutation [3]. Underlying triggers, such as infections, malignancy, or pregnancy, can also induce the clinical manifestation of aHUS. As more data from aHUS patients is reported, such principles are subject to change. Assessing the pathogenesis, triggers, manifestations, and treatment options for aHUS is vital for early diagnosis and management; however, recognizing previously unknown mechanisms, causations, and symptoms is equally as important. This review investigates the evolving clinical nature of aHUS in pediatrics, specifically focusing on triggers and emerging therapeutic options.

## 2. The Complement System

The complement system serves a critical role in the innate immune system with respect to three major physiological outcomes: membrane attack complex (MAC) formation, propagation of anaphylatoxins, and opsonization of pathogens. The three distinct pathways that launch the propagation of the complement system are the classical pathway (activated by a microbial-bound immunoglobulin G dimer), the lectin pathway (recognition of foreign particles via mannose binding lectin and ficolin), and the AP (continuously expressed and activated in the serum).

In aHUS, the AP is continuously active via hydrolytic activation of C3 into C3b by the ubiquitous water molecule and is regulated to prevent AP activated complement system attack on host cells. Regulatory factors found both in the serum and on the host cell surface function to inactivate C3b if not in the presence of a pathogen.

AP activated C3 protein is enzymatically divided into two parts: C3a and C3b. C3a serves as an anaphylatoxin while C3b further integrates into the complement pathway. C3b merges with activated complement factor B (Bb) to serve as a C3 convertase to further amplify inactivated C3 and can also recruit synthesized C3b to form the integral C5 convertase (schematically written as C3bBbC3b). The C5 convertase cleaves complement 5 (C5) into an anaphylatoxin (C5a) and the activated complement pathway mediator C5b. C5a increases the permeability of blood vessels and attracts inflammatory cells via chemotaxis. C5b further recruits complement factors 6, 7, 8, and multiple complement factor 9s to form the MAC, denoted as C5–9, ultimately killing the microorganism.

**Figure 1:**
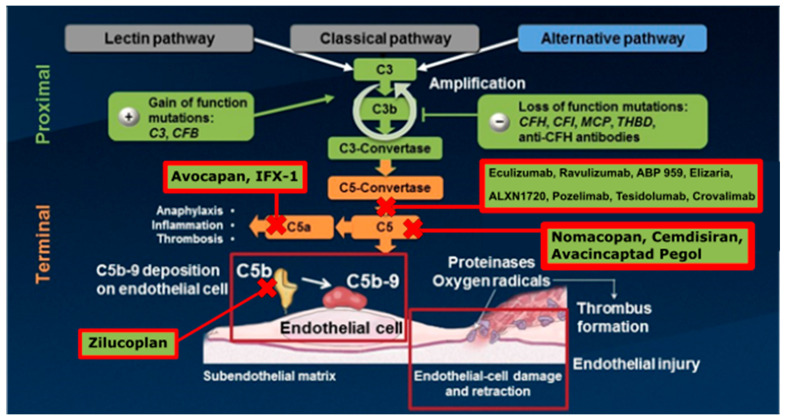
Alternative pathway with key emphasis on interventional drugs and where they act upon. Current therapeutics include Eculizumab, Ravulizumab, Avocapan, Nomacopan, and Cemdisiran. Biosimilars include ABP 959 and Elizaria. Drugs currently being developed include ALXN1720, Poselimab, Tesidolumab, Crovalimab, Avacincaptad Pegol, IFX-1, and Zilucoplan.

Atypical HUS, and the broader category of TMAs, are brought about by an imbalance of AP activated C3 protein and the regulatory factors deactivating it. Eculizumab, the typical course of treatment for aHUS, targets complement C5 by inhibiting the cleavage of C5, thus inhibiting MAC formation [3,4] (Figure 1).

## 3. Genetics

Since genetic abnormalities affecting the complement system are present in roughly 60% of patients diagnosed with aHUS, genetic testing is recommended for all potential aHUS patients under at least one of the following categories: tested negative for Shiga toxin-producing *Escherichia coli,* HUS after reporting prodromal diarrhea, have persistent thrombocytopenia, or reported ADAMTS13 activity levels <50% [1,2,3]. Genetic testing for heterozygous pathogenic mutations or polygenic mutations is primarily centered on C3, CD46, CFB, CFH, CFI, or thrombomodulin (THBD). A complete gene panel test should also include heterozygous or homozygous pathogenic variants of diacylglycerol kinase epsilon (DGKE), methylmalonic aciduria and homocystinuria, cobalamin C (MMACHC), C3, CD46, CFB, CFH, CFD, and CFI as well as homozygous deletions of the CFHR genes (typically CFHR1–3 and 5) [1,2,3]. An additional element to CFH and aHUS includes spontaneous mutations that lead to anti-complement factor H autoantibodies (anti-CFH) [5]. Homozygous deletions in CFHR1 and CFHR3 have been associated with the formation of anti-CFH antibodies, although the exact mechanism responsible is not yet known [6].

These guidelines for genetic testing are affirmed by a number of studies that have analyzed the data on complement system dysfunction mediated by genetic mutations and autoantibodies. Our meta-analysis over 12 studies (*n* = 2317) show 54.21% (*n* = 1256) of cases having at least one functional mutation in a complement gene (Table 1). The pooled proportion of CFH mutations was the highest at 21.41% (95% Confidence Interval (CI); 16.60–26.64%; *p* = 0.5189) and CFB was the lowest at 1.55% (95% CI; 0.99–2.32%; *p* = 0.7374). Other notable complement mutations also include C3 (5.29%), CFI (6.89%), THBD (1.74%), and CD46 (9.98%).

## 4. Triggers

**Figure 2:**
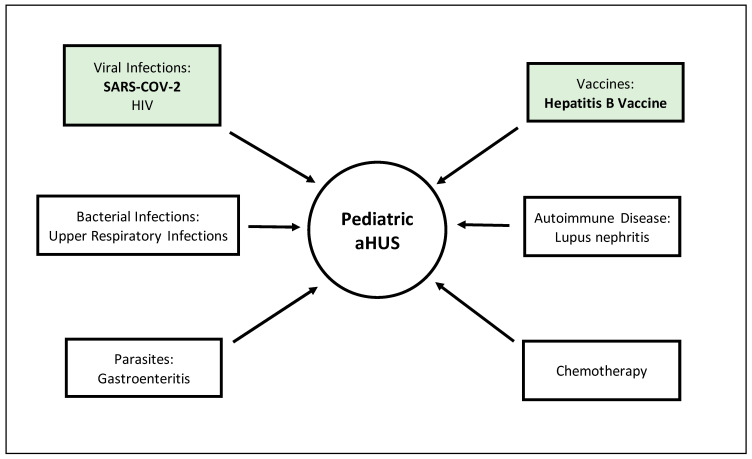
Atypical hemolytic uremic syndrome triggers. Novel developing triggers are highlighted in green. SARS-COV-2: severe acute respiratory syndrome coronavirus 2; HIV: human immunodeficiency virus.

Heterozygous mutations predisposing children to aHUS are often not sufficient to clinically manifest the disease due to poor penetrance—a trigger is often required. Tomazos et al. conducted a retrospective study (*n* = 147) identifying the primary triggers of adult aHUS as infection (63%, *n* = 92), chemotherapy (16%, *n* = 28), and systemic lupus erythematosus (15%, *n* = 26). Bacterial infections, primarily of the upper respiratory tract, predominated the infection subgroup at 42% (*n* = 75) [7]. More than one-third of all aHUS cases have unidentified triggers, which makes its sporadic presentation, especially in pediatrics, even more confounding. However, research identifying known causes, as well as novel ones, is rapidly developing (Figure 2) [8].

### 4.1. COVID-19

Emerging studies have shown a cyclical relationship between aHUS and COVID-19. TMAs, as well as the activation of interalveolar endothelial cells, in COVID-19 have led to the postulation that aHUS contributes to the pathogenesis of COVID-19. This relationship may be cyclical, where COVID-19 can be a contributing factor in the clinical manifestation of aHUS, even in patients who have recovered from the virus. SARS-CoV-2 and aHUS are both associated with venous thromboembolism, microvascular thrombosis, and multi-organ damage due to hyperactivation of the complement system [9]. SARS-CoV-2 binds to the angiotensin converting enzyme 2 (ACE-2) receptor in endothelial cells, causing the release of cytokines. The viral S glycoprotein binds to mannose associated serine protease (MASP2) and mannose binding lectin (MBL), leading to the activation of lectin and the AP [10]. The increased deposition of MBL, MASP-2, C3b, C4b, and C5b-9 on endothelial cells in COVID-19 patients is similar to the pathophysiology demonstrated in aHUS, which may serve to explain COVID-19’s potential triggering function of aHUS [11,12].

The underlying complement pathophysiology of COVID-19 is supported through the successful use of complement-mediated therapeutics. Cp40/AMY-101 is a compstatin-based C3 inhibitor developed by Amyndas Pharmaceuticals [13,14]. AMY-101 is suggested for patients with COVID-19 complicated by acute respiratory distress syndrome (ARDS), as it has shown positive results in alleviating symptoms in a COVID-19 patient [15]. BDB-100, also being tested to treat ARDS, was developed by the Beijing-based biopharmaceutical company Staidson. The anti-C5a antibodies of BDB-001 act to selectively block C5a without affecting the C5b pathway, thereby not affecting the membrane attack complex (MAC) formation. The administration of BDB-001 anti-C5a antibodies to treat two COVID-19 patients resulted in the elimination of fever, hepatic dysfunction, respiratory symptoms, and systemic inflammation. C5 inhibiting therapeutics, Eculizumab and Ravulizumab, used to treat aHUS patients were beneficial in combating COVID-19 in four patients [16]. The successful use of complement-mediated medications to treat COVID-19 further emphasizes the involvement of the complement pathway in COVID-19, and suggests a unique association between COVID 19 and aHUS which requires further investigation to confirm.

The relationship between COVID-19 and aHUS, primarily observed in adults, can also be extended to children. Mahajan et al. observed a 14-year-old female presenting with renal failure upon contracting SARS-CoV-2 [17]. This patient had notably increased inflammatory marker levels, elevated Cb5–9, and decreased levels of C3, indicating complement-mediated TMA. After successful treatment with three doses of Eculizumab, the patient’s renal function recovered, serum complement levels normalized, and the hematuria, proteinuria, and hypertension were all resolved. Alizadeh et al. reported a similar case of an infant male with no known genetic abnormalities who developed aHUS after contracting COVID-19 [18]. These reports suggest that COVID-19 has the potential to trigger the development of aHUS in pediatric patients.

COVID-19 is also postulated to act as a trigger for the manifestation of aHUS in the presence of pathogenic CFH variants. Kaufeld et al. observed two adult patients who contracted COVID-19 and developed thrombocytopenia, hemolytic anemia, and AKI; they were suspected to have aHUS and were diagnosed after genetic testing [19]. Further examination revealed that both patients had a pathogenic variant of CFH (c.3493 + 5G > A (chr1:g.196715134G > A) and c.2792G > A *p*.(Cys931Tyr)). The latter mutation was found to affect the tertiary structure of the CFH protein by interfering with a cysteine residue (CCP16c) involved in disulfide bridges, decreasing CFHs capability to regulate the complement system, which ultimately affects the protein structure. Although both patients described were adults, the genetic basis of these cases suggest that children with pathogenic CFH variants could face a similar manifestation, emphasizing the importance of genetic testing in pediatric patients prone to aHUS [19]. A list of novel triggers is highlighted in Table 2.

### 4.2. Hepatitis B Vaccine

Clinical manifestation of aHUS has also been reported after the hepatitis B vaccine. Avci et al. observed a 55-day-old infant female who exhibited aHUS-like symptoms one day after receiving the second dose of the hepatitis B vaccine, which contained 10 ug HBsAg/0.5 mL and 0.475 mg aluminum hydroxide/0.5 mL. This patient faced no complications after receiving the first dose of the vaccine at birth but developed jaundice and icteric sclera after the second dose. Notable findings included depressed hemoglobin levels and schistocytes as well as abnormal levels of total bilirubin, LDH, C3, and creatinine. A renal ultrasound also identified echogenicity in her kidneys. Consequently, the combination of acute renal failure, microangiopathy, hemolytic anemia, and thrombocytopenia led to the diagnosis of aHUS [20]. Similarly, Geerdink et al. reported on a patient who developed aHUS two days after receiving the hepatitis B vaccine [21]. Such cases suggest that the hepatitis B vaccine might be a trigger for pediatric aHUS.

## 5. Manifestations

As a TMA characterized by thrombocytopenia and microangiopathic hemolytic anemia, aHUS can induce organ damage due to ischemia downstream of occluded blood vessels [22,23]. Resulting extrarenal complications can present in aHUS patients and often affect the central nervous system (seizures, cortical blindness, encephalopathy, and drowsiness), pulmonary system (pulmonary hemorrhage), cardiovascular system (myocardial infarction, heart failure, and cardiomyopathy), gastrointestinal system (intestinal bleeding, pancreatitis), and the skeletal system (rhabdomyolysis). A list of clinical manifestations of aHUS based on organ system can be found in Table 3, with individual reports in Table 4.

### 5.1. Renal Manifestations

Lapeyraque et al. observed adult and pediatric aHUS patients (*n* = 22 and *n* = 15, respectively) and found that 100% of the patients reported renal manifestations at initial presentation and 68.4% during the chronic phase (study entry ≥6 months after initial presentation) [2]. This typically manifested as uremia. In general, most patients diagnosed with aHUS present with the typical aHUS triad (microangiopathic hemolytic anemia, thrombocytopenia, and AKI) as well as oliguria/anuria [24]. Potential life-threatening complications include hyperkalemia (≥6 mmol/L), acidosis (HCO_3_ < 15 mmol/L), and volume overload accompanied by arterial hypertension and hyponatremia (<125 mmol/L). The severity of arterial hypertension is exponentiated due to hyperreninemia secondary to renal thrombotic microangiopathy. The potential progression of aHUS in a patient can be estimated by the presence of acute renal failure, proteinuria, and/or hematuria as well as creatinine levels at the initial presentation [24,25].

### 5.2. Neurological Manifestations

Neurological complications have been reported in 8–48% of all aHUS patients, and more precisely up to 27.2% of pediatric aHUS patients [26,27]. Primary neurological symptoms include seizures, headache, altered consciousness, hemiparesis, vision loss, hallucinations, encephalopathy, agitation, confusion, reduced reflexes, hemiplegia, nystagmus, diplopia, focal neurologic deficits, and coma. Cerebral imaging in TMA patients shows changes in the posterior cortex and white matter, deep white matter, brainstem, basal ganglia, and thalami [28,29]. Several studies have reported that Eculizumab is effective in treating the neurological symptoms of aHUS. Guleroglu et al. observed two pediatric aHUS patients with neurological symptoms and irregular brain MRI results, which resolved after Eculizumab treatment [28]. Diamante et al. reported the successful resolution of multifocal hyperintensities and altered consciousness in a pediatric aHUS patient with anti-factor H antibodies [30]. Prompt management of electrolyte correction, urea reduction rate, blood pressure, and intracranial pressure effectively decreased the risk of chronic neurological symptoms. 

### 5.3. Ocular Manifestations

Reduced visual acuity, ocular pain, visual scotomas, diplopia, and blurred vision are often observed in aHUS patients. Pediatric ocular manifestations often include optic disc edema, bilateral flame-shaped intraretinal hemorrhage, and tortuosity. Zheng et al. observed an 11-year-old girl with aHUS who presented with decreased visual acuity [31]. In her right eye, what non-visually impaired people would see at 100 feet, she could only see at 20 feet (20/100). In her left eye, what non-visually impaired people would see at 200 feet, she could only see at 20 feet (20/200). Her additional symptoms included intraretinal hemorrhages, venous stasis retinopathy, and vein occlusions. She regained perfect visual acuity (20/20) three months after receiving fresh frozen plasma and red blood cell infusions—a staple aHUS treatment. 

### 5.4. Cardiovascular Manifestations

Cardiovascular complications are reported in 3–10% of all aHUS patients and affect 7% of pediatric aHUS patients [27]. Cardiac dysfunction can present due to AKI, hypertension, and/or associated fluid overload, as well as direct damage to the vasculature and cardiac tissue. Associated conditions include hypertrophic cardiomyopathy, left ventricular hypertrophy, elevated CK-MB levels, dilated cardiomyopathy, valve insufficiency, tachycardia, and intracardiac thrombus. Individuals with aHUS who have associated mutations in CFH, CFB, C3, and/or anti-factor H antibodies are susceptible to cardiovascular complications, estimated at 20% [32,33,34]. A 9-month female with aHUS, an individual from an Australian aHUS patient cohort, a 1-year-old girl with aHUS, and 10/23 children with diarrhea-negative HUS developed cardiomyopathy [32,33,34]. Steno-occlusive arterial disease has also been identified in pediatric aHUS patients, demonstrating the potential involvement of large arterial vessels. For example, the MRA of a 15-year-old aHUS patient with CFH mutation and ESRD showed middle and anterior cerebral artery stenosis [35,36]. Cardiac function has improved upon administration of Eculizumab, suggesting its effectiveness in treating cardiac complications in aHUS as well [32,34].

### 5.5. Pulmonary Manifestations

Johnson et al. reported that 21% of a cohort of 71 pediatric aHUS patients developed respiratory failure and required mechanical ventilation [37]. Pulmonary complications include pulmonary embolism, pulmonary hemorrhage, and pulmonary edema. 

### 5.6. Gastrointestinal and Dermatologic Manifestations

Besbas et al. observed a pediatric aHUS cohort and found 10% displaying gastrointestinal complications of vomiting, cholelithiasis, transaminitis, pancreatitis, hepatitis, and gastrointestinal bleeding [38]. Factors such as auto-CFH antibodies may put pediatric patients at greater risk for gastrointestinal complications, as Dragon-Durey et al. reported that more than 80% of patients with anti-CFH antibodies can have gastrointestinal symptoms [5]. Similarly, Roman-Ortiz et al. observed vomiting and abdominal pain in 56% of their pediatric aHUS cohort [39]. Web et al. also observed GI symptoms in a 16-year-old male with aHUS, which resolved after seven weeks of treatment [40]. In the context of dermatology, changes in the skin often present as a peripheral gangrene or cutaneous rash. In pediatric patients, changes to the peripheral vasculature primarily develop in the early course of the disease. Peripheral ischemia and changes in the skin respond positively to Eculizumab, emphasizing its broad therapeutic coverage [26].

## 6. Selected Mutations and Anti-Complement Factor Antibodies

### 6.1. Complement Factor H and Anti-Complement Factor H Antibodies

Preliminary reports have observed anti-CFH immunoglobulin G antibodies in 25–50% of pediatric aHUS cases [41]. aHUS mediated by anti-CFH antibodies has a 16% mortality rate and can cause kidney sequelae in around 50% of surviving patients [42]. This is observed through elevated anti-CFH antibody titers, which can rise to 1000–50,000 AU/mL [24]. CFH binding to platelets is a well-established mechanism, but information about the binding of platelets to anti-CFH antibodies requires further examination [43].

CFH regulates the AP of the complement system by directly binding to polyanions and compounds that activate C3. CFH, located near CFHR1 and CRFH5 genes, is composed of 20 short consensus repeat (SCR) domains, four of which are involved in the complement regulatory functions. Recent reports have demonstrated that the homozygous deletion of CFHR1 produces anti-CFH antibodies [44]. These antibodies bind to the N and C terminus of the CFH protein, decreasing CFHs ability to bind to C3b, C3c, and C3, resulting in the abnormal hemolysis associated with aHUS. In vitro binding of anti-CFH antibodies to CFH also causes lysis of platelets, which subsequently over-activates the complement system [43,44,45,46]. Anti-CFH antibodies have also been shown to interfere with the binding of CFH to glomeruli endothelial cells [46] and have been recently identified as a prognostic factor for aHUS in pediatric patients. 

aHUS due to anti-CFH antibodies was identified in a pediatric aHUS cohort (*n* = 436), with two cases of homozygous deletions of CFHR1-CFHR3 and anti-CFH autoantibodies in pediatric aHUS patients [45]. Both patients were successfully treated with Eculizumab and mycophenolic acid (MPA), as identified by stable anti-CFH antibody titer levels below <1000 UA. Notably, Matrat et al. is the first to report successful treatment of anti-CFH aHUS in pediatric patients with Eculizumab and MPA [42]. Previously, plasma exchange (PE), Rituximab, and cyclophosphamide have been widely used despite significant side effects, including cardiotoxicity, osteoporosis, decreased fertility, and an increased risk for tumors.

The exact site of anti-CFH binding to SCR has been debated, where Norris et al. and Wuo et al. report its binding at SCR 19–20, while Pursawani et al. reports its binding to the C-terminus of CFH at SCR 17–20 and SCR 5–8 [41,45,46]. Geographical differences have also been observed in the frequency of anti-CFH aHUS. These antibodies affect 5–25% of aHUS patients in Europe, but over 50% of aHUS patients in South Asia. Its prominence and prevalence in South Asia is thought to be due to higher rates of exposure to respiratory and GI pathogens, with the majority of anti-CFH antibody aHUS cases in South Asia among children ages 4–11 years old. Future research should seek to clearly define the true binding mechanism, which may explain the regional variations and contribute to the potential management and treatment of anti-CFH aHUS.

### 6.2. Complement Factor I and Anti-Complement Factor I Autoantibodies

CFI is a serum serine protease which cleaves C3b and C4b and prevents the formation of C3 and C5 convertase. Mutated forms make up 4–8% of aHUS cases, obstructing the secretion of proteins or altering CFI’s ability to cleave C3b and C4b, resulting in cleavage into abnormal products. This mutation affects both the cell surface and fluid phase, and in 20–30% of cases is associated with lower C3 levels [41].

Novel reports of CFI autoantibodies have recently emerged in a small subset of aHUS cohorts. Genetic testing of affected individuals revealed that all individuals with CFI autoantibodies have two copies of *CFHR1* and *CFHR3* genes, suggesting that CFI autoantibodies affect the development of CFH since low CFH levels were observed in all three patients. Govindarajan et al. reported CFI autoantibodies identified in 31% of a sample pediatric aHUS population in India [47]. Age notably affected which antibodies were the most common, where aHUS patients under 2 years old mainly had CFI autoantibodies and children over 2 years old had anti-CFH antibodies.

### 6.3. Diacylglycerol Kinase Epsilon 

The first non-complement system gene associated with aHUS was identified as DGKE and has been detected in 27% of all aHUS patients [48]. DGKE phosphorylates diacylglycerol to phosphatidic acid, which ultimately activates protein kinase C. The exact mechanism by which mutations in DGKE manifest in aHUS has not yet been identified; however, Zhu et al. hypothesized that the absence of DGKE may result in modifications of the vascular tone actin cytoskeleton, secretion of prothrombotic and antithrombotic factors, and the activation of platelets. These changes were confirmed when DGKE knockout mice showed irregularities in the extracellular matrix basement membrane and glomerular endothelium, occluded glomerular capillaries upon exposure to nephrotoxic serum, and impaired synthesis of prostaglandin E2 and cyclooxygenase 2 [49]. Lemaire et al. reinforced Zhu et al.’s reports of DGKE mutations clinically manifesting as chronic TMA characterized by glomerular cellularity, split glomerular basement membranes, swelling of endothelial cells, and widening of the glomerular basement membrane (GBM) internal lamina rara in the absence of electron-dense deposition [48].

Brocklebank et al. outlined the genotype, phenotype, and pathophysiology of DGKE mutations associated with aHUS in 16 patients (median age 9 months) [50]. The phenotype of DGKE mutations was characterized by persistent proteinuria, at least one relapse in children, and hypertension with hematuria leading to chronic kidney failure. Genetic analysis reported ten new missense mutations, both homozygous and heterozygous, yet the exact mechanism of how the DGKE mutations manifested is still not clear. The authors found a disproportionately high rate of developmental disorders in DGKE aHUS children. It was postulated that this could be due to DGKE’s function in controlling the signaling pathways of neurons in the brain. Brocklebank et al. reported no long-term clinical improvement after treatment with Eculizumab in aHUS patients with DGKE mutations and suggested refraining from administering Eculizumab to avoid the side effects of complement-inhibiting therapy [50]. As this study reported no relapses in transplant patients, the authors suggested renal transplantation for aHUS patients with DGKE mutations who reach ESRD. Due to relapses in aHUS patients with DGKE mutations on Eculizumab, the terminal pathway might not be directly involved. Reduced C3 levels have been observed in some aHUS patients with DGKE mutations, but whether it signals a bystander effect or the potential involvement of the complement system must be further examined.

## 7. Current Therapeutics

### 7.1. Immunotherapy

Plasma exchange (PE) and plasma infusions (PI) were once the generally accepted method of treating aHUS, despite little to no supportive clinical trials. PE allows for the relatively rapid removal of antibodies in autoimmune TMA and other malignant proteins, and can eliminate vWF multimers or autoantibodies. PE and PI function to alleviate the symptoms of TMAs or TPPs; however, they fail to accurately solve the root problem. After 2010, the guidelines were re-examined after unremarkable and even negative effects from PE and PI plagued many patients [37]. As the understanding and diagnostic capabilities regarding aHUS as grown within the last few decades, so have advances in therapeutics. However, it should be noted that less developed countries still rely on PE and PI due to costs and availability. Currently, there are three medications that are potentially usable for treating aHUS: Eculizumab, Ravulizumab, and Avacopan, all targeting and reducing the C5/C5a axis. 

Fremeaux-Bacchi et al. and Loirat et al. observed aHUS patients with CFH mutations and found that those who received high-intensity plasma therapy had similar outcomes regarding the progression of ESRD compared to those who did not [24,51]. In institutions where the anti-C5 inhibitor Eculizumab is not readily available, PE continues to be the first line of treatment [52]. Within 24 h of presentation, PE is typically initiated and encompasses daily infusions (1.5 times the plasma volume; 660–75 mL/kg) until platelet count, LDH, and hemoglobin levels are normalized [53]. If a patient’s hematological and renal functions are unresponsive to PE, Eculizumab is then required. The combination of PE and immunosuppressive agents, especially in patients with anti-factor H antibodies, has shown positive results primarily in adults in the literature [54,55,56]. However, its combined efficacy in the pediatric population is largely unknown due to the lack of randomized controlled trials.

### 7.2. Eculizumab

Eculizumab, a monoclonal antibody, was the first successful terminal complement inhibitor available to pediatric aHUS patients [57]. Eculizumab targets the complement system by binding to C5 and preventing its cleavage into C5a and C5b. This mitigates the proinflammatory effects of C5a, prevents the formation of C5b-9, and protects the functionality of upstream innate immune complement factors, such as C3a and C3b. [4,58]. Table 5 highlights four of the trials for Eculizumab.

Greenbaum et al. reported the first prospective phase II trial for pediatric aHUS patients in 2016 [59]. After 26 weeks, hematological parameters and platelet count were normalized in 82% of patients (median 55 days and 7 days, respectively), 73% had at least a 25% decrease in serum creatinine levels, and 86% had improvements in eGFR levels, all signifying improved renal function. All patients were able to discontinue PE/PI and 82% of patients were able to discontinue dialysis with no resulting deaths or meningococcal infections. Despite the frequent doses needed at least twice a month, overall quality of life improved after 26 weeks of treatment, which supports the use of Eculizumab in pediatric aHUS patients. After one year of PE/PI use, nearly a third of patients typically died or developed ESRD; thus, the development of Eculizumab has significantly improved patient outcomes.

Ariceta et al. contrarily observed 90.9% and 59% of children on eculizumab experience adverse side effects (cough, fever, abdominal pain, respiratory tract infections, and diarrhea) and serious complications (elevated severity of the aforementioned), respectively [60]. Eculizumab is ineffective in patients with polymorphic forms of C5, as it is unable to bind to C5 variants; hence, it is unable to block the cleavage of C5 in patients. Due to a greater risk of infection from encapsulated bacteria, such as meningococcal infections, pediatric aHUS patients require prophylactic vaccinations [58,61]. Eculizumab is commonly used today for the treatment of aHUS, and has paved the way for the development of other aHUS therapeutics such as Ravulizumab. A list of current, developing, and future therapeutics can be found in Table 6.

### 7.3. Ravulizumab

Ravulizumab is a humanized monoclonal antibody developed by the same manufacturer as Eculizumab. Similarly, it acts as a C5 inhibitor by binding with greater specificity and affinity to C5 to prevent the terminal complement complex formation. This second-generation drug was developed by modifying Eculizumab to create a novel, longer-acting antibody which would require less frequent infusions than its predecessor. The drug was developed through the Xencor antibody half-life prolongation technology Xtend; a technology that increases half-life through Fc variants [62]. Through modifications in amino acids, this drug enhances the rate at which the mAb:C5 complex dissociates in the acidic early endosome at pH 6.0 and increases antibody recycling of the neonatal Fc receptor, both of which increase the life of terminal complement inhibition. Ravulizumab has a half-life 4-fold greater than that of Eculizumab, allowing patients to receive infusions every 2–8 weeks rather than bi-monthly, as with Eculizumab [58].

Tanaka et al. demonstrated the successful and safe use of Ravulizumab in pediatric patients (*n* = 10) who were previously being treated with Eculizumab [63]. Each patient received doses on day 1, day 15, and subsequently every 4–8 weeks. During the clinical trial, no patient required dialysis and kidney function, platelet count, hemoglobin, LDH levels, and inhibition of the terminal complement pathway all remained stable. Ehren et al. conducted a similar study where patients (*n* = 6) who were previously treated with Eculizumab were given Ravulizumab [64]. All patients were found to have had stable renal and hematological parameters as well as consistent suppression of AP50 and sC5b-9 levels for three months. No side effects were reported after switching to Ravulizumab, and every patient reported an enhanced quality of life because of the longer dose intervals. Ehren et al. reinforced the findings of Tanaka et al. and added further evidence that Ravulizumab is safe and successful in the treatment of pediatric aHUS patients who were previously treated with Eculizumab.

Ravulizumab has also been analyzed in pediatric aHUS patients who have not been treated with complement inhibition therapy. Ariceta et al. described the first prospective phase III trial which demonstrated that Ravulizumab is safe and successful in treating complement inhibitor-naïve pediatric patients [60]. Patients less than 20 kg were given infusions every 4 weeks and patients more than 20 kg were given infusions every 8 weeks. This trial led to complete inhibition of C5, causing normalization of hematological parameters and repaired renal function. A complete TMA response was characterized by the normalization of platelet count and LDH, and a ≥25% increase in serum creatinine levels. Of the total participants, 77.8% attained this response by 26 weeks, and a total of 94.4% patients reached a full TMA response by week 50. Platelets normalized faster than other TMA response components, 8 days after the first infusion. All participants had either discontinued or were never started on dialysis by week 50, and each patient had improved renal function by week 50 (measured via eGFR). More importantly, all participants had improved FACIT-fatigue scores at 26 and 50 weeks, which is suggestive of an improved risk-benefit ratio for Ravulizumab treatment.

The major limitation to using Ravulizumab is that its affinity for C5 is 17-fold greater than Eculizumab, which ultimately stunts the immune system and makes patients susceptible to infection [58]. Tanaka et al. observed recurring oropharyngeal pain (30%) and upper respiratory tract infections (40%) in pediatric participants in their study [63]. Ariceta et al. reported that 47.6% of participants in their study had drug induced complications, including pyrexia, nasopharyngitis, vomiting, diarrhea, and headaches [60]. However, both studies concluded that the benefits outweighed these risks. Ariceta et al. reported no cases of meningococcal infections and less frequent infusions (decreasing from 26 to 7–13 infusions per year). Additionally, aHUS patients may not need a port to access the vasculature, preventing related comorbidities from infections or clots. Patients on Ravulizumab require less appointments, which may decrease potential exposure to nosocomial infections. Children may also experience less fear, pain, and illness from the venipuncture. These potential benefits are indicative of higher quality of life for pediatric aHUS patients on Ravulizumab. The improved efficiency of Ravulizumab allows for 60% and 73% lower productivity costs in the clinic and at home, respectively, also demonstrating decreased societal and healthcare-associated costs of therapy.

Similar in effect, Nomacopan, Cemdisiran, and Avacopan are three novel drugs that are currently being investigated in aHUS patients; however, they have yet to be established as primary treatments for aHUS, such as Eculizumab and Ravulizumab. Avacopan is an antagonist to C5aR1 that inhibits the functions of the C3a, C4a, and C5a protein [65]. It has demonstrated positive results in mice with glomerulonephritis, monkeys with neutropenia, and in Phase II and III trials of patients with AAV. A phase III adeno-associated virus (AAV) trial revealed Avacopan had significantly improved outcomes, including renal function, compared to other medications which have been studied after 26 and 52 weeks. Unfortunately, a different phase II trial initiated on six aHUS patients was terminated without a reported reason [58]. Nomacopan is a recombinant protein derived from a tick C5 inhibitor that binds to and inhibits C5 and leukotriene B4. It has been studied in aHUS patients, yet due to its 10 h half-life, daily subcutaneous injections would be required [58]. Kuhn et al. and Miles et al. suggest that an N-terminal fusion tag (PASylation) could perhaps increase the half-life of Nomacopan without reducing the efficacy of C5 inhibition, reducing the number of injections to once per week [66]. Cemdisiran consists of short sequences of interfering RNA, which match the mRNA for the C5 protein, with N-acetylgalactosamine. Upon its weekly or biweekly administration to monkeys, Cemdisiran stopped C5 production and decreased hemolytic activity to 80% [67]. This drug is now being studied in aHUS patients..

### 7.4. Kidney Transplant Setting

In patients with aHUS and a mutation in the complement factor H gene (*CFH*), there is an 80% risk of renal allograft failure within the first two years following the transplant [68,69]. Despite prophylactic PE, patients carrying the *CFH* or *CFH/CFHR1* hybrid genes have shown an increased risk of recurrences. Preemptive therapy in the form of Eculizumab has been positively associated with a decreased risk of recurrence, as reinforced by several case reports and controlled trials [70,71,72,73,74,75]. Multiple reports regarding the pediatric population have emphasized this anti-C5 drug’s efficacy in preventing post-transplant aHUS recurrence, especially for those at high risk [76,77,78,79]. Zuber assessed nine aHUS renal transplant patients all carrying a genetic abnormality associated with a high risk of recurrence and reported that 8/9 patients experienced a successful recurrence-free posttransplant course with the use of Eculizumab [73]. Ardissino et al. compared aHUS patients among three groups: (1) patients who underwent kidney transplantation without any preventative measures, (2) patients who received large volumes of FFP, and (3) patients treated with Eculizumab prophylactically. The use of Eculizumab resulted in the prevention of relapses compared to the other groups, with zero relapsing events compared to 0.81–3.1 events in the control over 10 years [80]. Similarly, Siedlecki et al. reported enhanced kidney function in the context of GFR in aHUS patients treated with Eculizumab both prophylactically and post-transplant (median 60.6 mL/min/1.73 m^2^) compared to those only treated with Eculizumab post-transplant (median 31.5 mL/min/1.73 m^2^) [81]. The risk of dialysis after transplantation was significantly higher in the latter group, with a hazard ratio (HR) of 4.6 (CI 1.7–12.4) compared to the former with an HR of 2.3 (CI 0.9–6.2). Despite the positive literature in regards to prophylactic Eculizumab treatment pre-transplantation, clarity is still required in regards to the schedule, dosage, and possibility of discontinuation [81].

### 7.5. Biosimilars

A biosimilar is a drug that is not significantly different from an established FDA approved drug with regards to its structure, pharmacokinetics (PK), pharmacodynamics (PD), purity, mechanism, safety, potency, and immunogenicity.

ABP 959, a biosimilar to FDA-licensed Eculizumab, was tested in healthy adult male subjects in a Phase III trial by Chow et al. for its safety and efficacy [82]. Participants aged 18–45 were given 300-mg intravenous (IV) infusions of the drug. The results of this study showed PK similarity between ABP 959 and Eculizumab, as measured by the PK parameters AUC0, AUClast, and Cmax. Measured by ABEC for CH50, PD between Eculizumab and ABP 959 were also similar. The ABP 959 infusions were deemed safe, as the occurrences of infections were similar in both groups; the reported grade 3 viral infection was concluded to be unrelated to the drug. Additionally, no participants developed neutralizing antibodies and the anti-drug antibody binding was similar in both ABP 959 and Eculizumab. Overall, Chow et al. demonstrated the safety and efficacy of the biosimilar ABP 959, as shown by similar PK and PD parameters and immunogenicity and safety profiles. 

Elizaria is the first Russian biosimilar to Eculizumab. It is used today in clinical practice, as phase III trials demonstrated similar safety, efficacy, PK/PD parameters to treat paroxysmal nocturnal hemoglobinuria, and immunogenicity as Soliris. The authorization of Elizaria in the market led to a 25% reduction in the cost of aHUS treatment, which expanded access to complement-inhibiting therapies for aHUS patients. Lavrishcheva et al. reported a case of a 46-year-old aHUS patient who was initially taking traditional Eculizumab and switched to Elizaria [83]. The drug demonstrated efficacy, as this patient had improved renal function, decreased proteinuria, and normalized blood pressure levels. This case also demonstrated drug safety, as no serious adverse events were noted in the follow-up period, and discontinuation of the trial was not necessary. Although this study describes the case report of an adult, it points to future studies which could potentially show the safety and efficacy of Elizaria in pediatric aHUS patients as well.

## 8. Future Therapeutic Options

A number of drugs utilized in other complement mediated disorders could potentially have therapeutic potential in aHUS. 

ALXN1720: The same manufacturer of Eculizumab and Ravulizumab is conducting phase I trials on an anti-C5 mini body that binds to the C5 protein and thereby blocks its activation [58].

Pozelimab: Pozelimab is a C5 antibody that has been shown to decrease hemolysis and C5 levels in both mice and human serum models more significantly than Eculizumab and Ravulizumab, and has shown successful phase II trial results in patients with PNH [58,84].

Tesidolumab: Tesidolumab is a C5 monoclonal IgG1 antibody that was initially developed to treat age-related macular degeneration, in which C5a and C5b deposition in Bruch’s membrane and retinal cells is observed. Novartis administered phase II studies of this drug on PNH patients [58,85].

Crovalimab: Crovalimab employs similar antibody recycling as does Ravulimab, increasing its half-life compared to Eculizumab [86]. Crovalimab binds to a different C5 epitope than does Eculizumab, and thus neutralizes C5 variants that Eculizumab cannot bind to. Fukuzawa et al. [87]. Additionally, Croalimab binding to C5b has also been also observed, preventing the formation of the MAC complex. This drug has a 90% bioavailability, which reduces the burden on patients as it can be self-administered. Phase I and II studies have been conducted in patients with PNH; administration of dosing every 1–4 weeks and interconverting with Eculizumab was shown to be safe [58,88].

IFX-1: IFX-1 targets the C5a protein directly and, consequently, does not affect the cleavage of C5 and the formation of C5b-9 [89]. Phase IIa trials have shown positive results in the skin disorders hidradenitis suppurativa and pyoderma gangrenosum. Currently, IFX-1 is being studied for the treatment of AAV and as a potential substitute for corticosteroid therapeutics. One study demonstrated reduced systemic inflammation and acute lung injury in monkeys with the H7N9 virus upon administration of IFX-1, and this drug is now being administered to COVID-19 patients exhibiting pneumonia in phase II and III trials [58].

Zilucoplan: This drug binds to the C5b protein and the C5b part of C5. This drug has been successful in phase II trials in generalized myasthenia gravis and underwent re-development after being tested in PNH patients [90]. This drug is now being tested as a treatment for COVID-19 [61].

Avacincaptad Pegol: Avacincaptad pegol binds to and inhibits the C5 protein, and is administered every month as a treatment for complement-mediated eye diseases, such as age-related macular degeneration and Stargardt macular dystrophy [58,91].

Avdoralimab: Avdoralimab is an anti-C5aR1 antibody that suppresses the C5aR1 function of blocking the activity of T-cells and natural killer cells in the presence of tumors [Dema]. Phase I studies testing this drug on patients with progressed solid tumors on PD-1 therapy were successful, showing complete blockage of C5aR1. Currently, phase II trials on patients with COVID-19 are being conducted [58].

MAC Inhibitor: HMR59: HMR59 is a gene therapy that enhances the synthesis of the C9 regulator in retinal cells, CD59, which blocks C5b-9. Phase I trials in AMD patients were conducted and phase II trials are underway [58].

Infusion of recombinant complement regulatory proteins is another developing technique. CFH would first be purified from human plasma fractions in order to supply a pathogen-safe and functional CFH. In theory, a mutational variant of CFH in aHUS could be treated with the administration of a purified CFH. This could be less expensive than PE and would avoid the use of a central venous line, which can cause infection; however, clinical trials have yet to be conducted [92].

The use of immunosuppressive agents, such as corticosteroids, decreases inflammation and provides symptomatic improvement; however, long-term use is associated with serious adverse events, of which the negative consequences outweigh its benefits. Complications with non-specific immunosuppressive agents include infection, diabetes, osteoporosis, weight gain, and hypertension [58]. Researchers continue to use the global registry of aHUS patients and novel case studies to improve their understanding of this disease.

## 9. Conclusions

The diagnosis and management of aHUS has drastically shifted over the past twenty years due to a number of scientific breakthroughs. Additional genetic variations and potential triggers continue to be identified as well as novel detections in autoantibodies to CFH and (more recently) CFI. Interventional management has seen the most growth, where less than ten years ago only PE/PI and Eculizumab were available. Currently, Eculizumab remains the standard of care; however, there are a number of therapeutic options that will soon become available, all with differing mechanisms of action depending on the exact mutational-based pathophysiology. As data continues to be evaluated, advances in the diagnosis and management of aHUS will continue to lower the associated morbidity and mortality rate in both adults and pediatric patients.

## Figures and Tables

**Table 1:** Pooled proportion of different mutations in aHUS patients.

Mutation	No. of Studies	Total aHUS Patients	Pooled Estimate	I^2^ (95% CI); *p* Value	Egger’s Test
			(95% CI)		
CFH	12	2295	21.41%	85.84% (76.97–91.29%);	*p* = 0.5189
			(16.60–26.64%)	*p* < 0.0001	
CD46	11	2177	9.98%	76.84% (58.58–87.05%);	*p* = 0.2614
			(7.15–13.22%)	*p* < 0.0001	
CFI	12	2295	6.89%	64.69% (34.6–80.93%);	*p* = 0.2206
			(5.01–9.05%)	*p* = 0.0010	
DGKE	4	558	6.57%	90.06% (77.48–95.61%);	*p* = 0.1619
			(0.93–16.76%)	*p* < 0.0001	
C3	9	2193	5.29%	61.37% (20.05–81.34%);	*p* = 0.8866
			(3.74–7.09%)	*p* = 0.0080	
THBD	6	1176	1.74%	77.9% (51.11–90.01%);	*p* = 0.6401
			(0.47–3.8%)	*p* = 0.0004	
CFB	5	1469	1.55%	26.03% (0.00–70.5%);	*p* = 0.7374
			(0.99–2.32%) *	*p* = 0.2480	
Others	4	691	19.29%	98.7% (97.98–99.16%);	*p* = 0.7916
			(1.34–50.78%)	*p* < 0.0001	
Combined	7	1922	3.06%	84.36% (69.48–91.98%);	*p* = 0.0566
			(1.26–5.61%)	*p* < 0.0001	

* Random effect model; for others the fixed effect model was used. ^ Data based on a single study, pooled estimate is not possible. Pooled estimate of proportion of aHUS patients was calculated with random effects model for high heterogeneity and fixed effects model for low heterogeneity. I^2^ test assessed the degree of between-study heterogeneity, where I^2^ ≥ 50% indicated high heterogeneity. Egger’s test for publication bias, where *p* < 0.05 indicted the presence of publication bias.

**Table 2:** Triggers to aHUS.

Novel Triggers to aHUS						
	COVID-19				Hepatitis B Vaccine	
	Mahajan et al.	Alizadeh et al.	Kaufeld et al.		Avci et al.	Geerdink et al.
Year	2020	2021	2021		2013	2012
Sample Size	1	1	1	1	1	1
Age (Months)	14	1.3	22	52	0.15	-
Symptoms at Onset	Abdominal pain Diarrhea Vomiting Myalgias Chest pain	Fever Tachycardia Tachypnea Metabolic Acidosis	Diarrhea Vomiting Loss of taste Hypertension	Loss of taste Fever Abdominal pain Hypertension	Jaundice Pulse rate: 140/min Respiratory rate: 48/min BP: 70/40 mmHg	x
Lab Results	↑Ferritin >100,000 ng/mL, ↑CRP > 300 mg/dL, WBC 33,000/µL SCr 0.7 mg/dL ↓Platelets 126,000/mL ↓Hgb 6.8 g/dL ↑LDH 4087 U/L ↑Bilirubin 6.2 mg/dL Schistocytes ↓C3 33 mg/dL ↓C4 1.0 mg/dL	↓Bicarbonate 4 mmol/L ↓PC02 17 mmHg ↓Anion gap 40 ↑Glucose 805 mg/dL ↑WBC 33,000 ↑Procalcitonin 3 ng/mL	↑LDH 2066 U/L ↓Hgb 5.5 g/dL	↑LDH 88,560 U/L ↓Hgb 9.4 g/dl	↓Hgb 51 g/L Leukocyte count 10 × 10^9^/L Platelet count 28 × 10^9^/L ↑Reticulocyte level 3.9% ↑Urea 88 mg/dL SCr 1.1 mg/dL ↑Total bilirubin 13.7 mg/dL ↑Direct bilirubin 2.6 mg/dL Uric acid 8.1 mg/dL ↑Asp aminotransferase 96 U/L ↑LDH 4641 U/L	x
Progressing Lab Results	↑SCr 8.97 mg/dL ↑BUN 170 mg/dL	↓Platelets <100 K/uL ↑Reticulosytosis 13% ↑LDH 3190 U/L ↑Bilirubmin 1.5 mg/dL ↑Fibrinogen 557 mg/dL ↑Ferritin 1493 ng/mL ↑BUN 39 mg/dL ↑Creatinine 0.39 mg/dL C3 142 mg/dL C4 19 mg/dL	↑SCr 6.33 mg/dL ↓Platelets 28 k cells/uL	↑SCr 2.88 mg/dL ↓Platelets 128 k/uL	x	x

Novel triggers to aHUS, presenting case studies with symptoms at onset, admission lab results, and developing lab results throughout the hospital stay. BP: blood pressure; CRP: complement reactive protein; SCr: serum creatinine; Hgb: hemoglobin; LDH: lactase dehydrogenase; C3: complement factor 3; C4: complement factor 4; BUN: blood urea nitrogen; PC02: partial pressure of carbon dioxide.

**Table 3:** Clinical manifestations of aHUS based on organ system.

Organ System	Clinical Manifestations		Reported Efficacy of Eculizumab
Renal	Glomerular thrombotic microangiopathy, Arterial TMA, and Cortical necrosis		Yes
Neurological	Seizures, Headache, Altered consciousness, Hemiparesis, Vision loss, Hallucinations, Encephalopathy	Agitation, Confusion, Reduced reflexes, Hemiplegia, Nystagmus, Diplopia, Focal neurologic deficits, Coma	Yes
Pulmonary	Pulmonary embolism, Hemorrhage, Edema, Respiratory failure		N/A
Dermatologic	Peripheral gangrene, Ischemia, Cutaneous rashes		Yes
Cardiovascular	Hypertrophic cardiomyopathy, Left ventricular hypertrophy, Elevated CK-MB level, Dilated cardiomyopathy, Valve insufficiency	Tachycardia, Intracardiac thrombus, Steno-occlusive arterial disease in large arterial vessels (i.e., middle and anterior cerebral artery stenosis)	Yes
Ocular	Reduced visual acuity, Ocular pain, Visual scotomas, Diplopia, Blurred vision	Optic disc edema, Bilateral flame-shaped intraretinal hemorrhage, Tortuosity, Venous stasis retinopathy	Yes
Gastrointestinal	Vomiting, Cholelithiasis, Transaminitis, Pancreatitis,	Hepatitis, Gastrointestinal bleeding, Abdominal pain	Yes

Clinical manifestations of aHUS based on organ system and documented effectiveness of Eculizumab.

**Table 4:** Reports of organ system complications in aHUS.

Organ System Complications Due to aHUS					
Organ System	Authors	Year	Sample Size	Age	Outcome
Neurological	Gulleroglu et al.	2013	2	14	Neurologic symptoms and irregular cerebral MRI results
	Diamante et al.	2014	1	<18	Multifocal hyperintensities and altered consciousness
Cardiovascular	Hu et al.	2015	1	0.75	Cardiomyopathy and altered cardiac function
	Vilalta et al.	2012	1	1	
	Neuhaus et al.	1997		23	
	Davin et al.	2010	1	15	Middle and anterior cerebral artery stenosis
Pulmonary	Johnson et al.	2014	71	-	21% of aHUS patients developed respiratory failure
Ocular	Zheng et al.	2014	1	11	Decreased visual acuity (20/100 in the right eye, 20/200 in the left eye) Intraretinal hemorrhages, Venous stasis retinopathy, and Vein occlusions
Gastrointestinal	Besbas et al.	2017	146	-	10% displayed vomiting, cholelithiasis, transaminitis, pancreatitis, hepatitis, and GI bleeding
	Dragon-Durey et al.	2010	45	-	<80% of patients with anti-CFH antibodies had GI symptoms
	Roman-Ortiz et al.	2014	1	9	Abdominal pain

Reports of organ system complications in atypical hemolytic uremic syndrome patients/cohorts. MRI: magnetic resonance imaging; GI: gastrointestinal; CFH: complement factor H.

**Table 5:** Eculizumab treatment trials and resulting outcomes.

Eculizumab Treatment					
	**Legendre et al.**	**Licht et al.**	**Cofiell et al.**	**Greenbaum et al.**	
Year	2013	2014	2014	2015	
Sample Size	37	37	41	22	
Age (Years)	≥12	≥18 + 1 adolescent	≥18	0.4–17	
Primary Endpoints	Platelet normalization (≥150 × 10^9^/L) LDH normalization (<upper limit of normal) eGFR via SCr improved (≥25% reduction from baseline)				
Secondary Endpoints	26 weeks = 80%	26 weeks = 82%	-	27 weeks = 95%	TMA free outcomes
	26 weeks = 100%	26 weeks = 76%	-	55 days (median) = 82%	Hematologic normalization
	26 weeks = 100%	-	6 weeks = sig. ↓	-	Complement pathway inhibition
	26 weeks = 100%	26 weeks = 80%	6 weeks = sig. ↓	48 days (median) = 73%	Renal function measure normalization

Early Eculizimab trials and resulting outcomes. LDH: lactate dehydrogenase; SCr: serum creatinine; L: liter; eGFR: estimated glomerular filtration rate; TMA: thrombotic angiopathy anemia.

**Table 6:** Current, developing, and future therapeutics for aHUS.

	Current Therapeutics	Drug Class	Pathophysiology/ Mechanism of Action	Complement Pathway Proteins Affected
Current Therapeutics.	Eculizumab	Monoclonal Antibody, terminal complement inhibitor	Binds to C5 and prevents cleavage to C5a and C5b	C5a and C5b levels
	Ravulizumab		Prevents the cleavage of C5 into C5a and C5b	
	Nomacopan	C5aR1 antagonist	Inhibits C3a, C4a, and C5a protein function	C3a, C4a, and C5a levels
	Avocapan	Recombinant protein derived from a tick C5 inhibitor	Inhibits C5 and leukotriene B4	C5 and leukotriene B4 levels
	Cemdisiran	Short sequences of interfering RNA	Match mRNA for the C5 protein, with N-acetylgalactosamine	C5 levels
Biosimilars	ABP 959	Biosimilar to FDA-licensed Eculizumab	Binds to C5 and prevents cleavage to C5a and C5b	C5a and C5b levels
	Elizaria	Russian biosimilar to Eculizumab	Binds to C5 and prevents cleavage to C5a and C5b	
Future Therapeutics	ALXN1720	Anti-C5 mini body	Binds to C5 protein and blocks its activation	C5 levels
	Pozelimab	C5 antibody	Decrease hemolysis and C5 level	
	Tesidolumab	C5 monoclonal IgG1 antibody	Binds to C5 preventing its cleavage	C5a and C5b levels
	Crovalimab	Binds to a C5 epitope	Binds to C5b and prevents the formation of the MAC complex	C5a, C5b, and MAC complex proteins
	IFX-1	Targets C5a protein directly	Binds to C5a	C5a levels
	Zilucoplan	Binds to the C5b protein and the C5b part of C5	Inhibits C5b binding on C5 by binding to its C5 domain	C5b levels
	Avacincaptad Pegol	Binds to and inhibits the C5 protein	Prevents cleavage of C5	C5 levels
	Avdoralimab	Anti-C5aR1 antibody	Blocks T-cell and natural killer cell activity through C5aR1 suppression	C5aR1 levels
	MAC Inhbitor HMR59	Promotes CD59 production	Enhances synthesis of CD59, which blocks C5b-9 formation	C5b-9 formation

List of current, developing, and future therapeutics for atypical hemolytic uremic syndrome.

## Data Availability

All data generated or analyzed during this study are included in this article. Further enquiries can be directed to the corresponding author.
